# Comprehensive Assessment of Triggers for Behaviours of Concern Scale (CATS): Initial Development

**DOI:** 10.3390/ijerph182010674

**Published:** 2021-10-12

**Authors:** Bharati Limbu, Gemma Unwin, Shoumitro (Shoumi) Deb

**Affiliations:** 1 Division of Psychiatry, Imperial College London, 2nd Floor Commonwealth Building, Du Cane Road, London W12 0NN, UK; s.deb@imperial.ac.uk; 2 School of Psychology, University of Birmingham, 52 Pritchatts Road, Room 314, Edgbaston, Birmingham B15 2TT, UK; g.l.unwin@bham.ac.uk

**Keywords:** challenging behaviours, behaviours that challenge, triggers of challenging behaviour, triggers for challenging behaviour scale, contextual assessment of challenging behaviour, intellectual disabilities

## Abstract

Challenging behaviour displayed by people with intellectual disabilities (ID) can be difficult to manage if caregivers do not understand the reasons for the behaviour. Identifying the contextual variables/triggers for the behaviour is likely to help undertake a functional analysis leading to a person-centred positive behaviour support plan. Currently, a limited number of checklists are available for trigger assessment and none were developed using an interview with the family caregivers. This article describes the development and contents of the comprehensive assessment of triggers for behaviours of concern scale (CATS). CATS was developed in two stages. Stage 1 used a ‘bottom-up’ approach, in which caregivers of adults with ID who show aggressive behaviour were interviewed to identify the triggers for aggression. In stage two, using a ‘top-down’ approach, a comprehensive literature review was conducted to gather items from existing trigger checklists. Trigger items from both stages were combined and the duplicates were removed. The final list in CATS consists of 333 contextual triggers categorised under five main domains and 12 subdomains. CATS can be used by caregivers to identify triggers or antecedents of challenging behaviour. Further work is needed to test its psychometric properties, utility, and acceptability.

## 1. Introduction

Challenging behaviour, also known as behaviour that challenges or behaviour of concern, is defined as behaviour that is of such an intensity, frequency, or duration as to threaten the quality of life and/or the physical safety of the individual or others; it is likely to lead to responses that are restrictive, aversive, or result in exclusion [1]. Challenging behaviour is common in people with intellectual disabilities (ID) with up to 60.4% adults with ID showing at least one form of challenging behaviour [2]. The prevalence of severe challenging behaviour among people with ID is between 10% and 18% [2,3]. This increases with the severity of ID, with up to 82% of people with multiple and profound disabilities displaying challenging behaviour [4]. Challenging behaviour has been reported in the form of aggressive, self-injurious (SIB), and destructive behaviour, and other forms of behaviours such as temper tantrum, overactivity, inappropriate sexual behaviour, night-time disturbance, etc. [2,3]. Challenging behaviour has detrimental effects on the person’s quality of life and their physical, social, and emotional wellbeing [5]. Challenging behaviour also incurs a significant cost [6] and has been associated with caregiver’s stress, exclusion from community facilities, and inappropriate and increased use of restrictive practices, including use of psychotropic medication and, in some cases, loss of community placement, leading to hospital admission [7,8]. Therefore, to reduce costs and improve the quality of life of the person with ID and their caregivers, challenging behaviour needs to be assessed effectively to guide appropriate interventions. The challenging behaviour is the outcome of a complex interplay among biological (physical illness, genetic syndromes, underlying brain damage, etc.), psychological (emotional trauma, abuse, stress, psychiatric disorders, etc.), and social/environmental (crowded environment, lack of personal space, etc.) conditions (see Appendix A). Therefore, a biopsychosocial approach is needed to formulate and implement an effective person-centred intervention by a multidisciplinary team.

It is proposed that challenging behaviour serves a function, and it is important to know that function to help ameliorate the behaviour [1,9]. Functional assessment is a well-known procedure to detect the function of the behaviour by identifying antecedents and consequences that maintain challenging behaviour [10]. This helps to develop a person-centred positive behaviour support plan for individuals with ID. Matson and colleagues [11] developed the questions about behavioural function (QABF) scale to aid functional analysis. The QABF categorises functions under six headings, namely attention (receive attention), escape (avoid something), nonsocial (factors internal to the person), physical (physical problems such as relief from pain), and tangible (achieve something). Function of challenging behaviour is strongly influenced by context, specifically contexts that involve physical, activity/routine, social, and biological variables [12,13,14]. Contextual variables can be considered as triggers to challenging behaviour or antecedents and have a functional relationship to challenging behaviour. For instance, if the function of challenging behaviour is to achieve attention from a particular support staff, then the absence of that support staff can increase instances of challenging behaviour. Presence or absence of support staff therefore acts as a specific contextual trigger to challenging behaviour. Biological/physical/genetic, psychological/psychiatric, and social/environmental contextual variables or triggers for challenging behaviours can be easily detected by untrained caregivers through the use of indirect assessments [10]. Triggers could be predisposing (e.g., genetic disorder) or precipitating (e.g., pain in the body) or perpetuating (e.g., crowded environment).

Five scales are available for the assessment of contextual variables, namely (a) contextual assessment inventory for problem behaviour (CAIPB) [13,14], (b) setting event inventory (SEI) [15], (c) setting event checklist (SEC) [16], (d) setting events list (SEL) [17], and (e) challenging behaviour attributions scale (CHABA) [18]. In addition, there are also six scales available for the assessment of function/motivation of challenging behaviour (see Table 1). Further details of the five contextual scales are presented in Table 2. However, none of these scales were developed using a ‘bottom up’ approach in which both family and paid caregivers were interviewed to detect triggers for challenging behaviour among people they care for. Therefore, we have developed the comprehensive assessment of triggers for behaviours of concern scale (CATS) for caregivers to use to detect triggers for challenging behaviour using a combined ‘bottom up’ (interview with caregivers) and ‘top-down’ (reviews of items in the existing scales) approach. This was done in the context of a larger project that developed a short-term psycho-education for carers to reduce over-medication of people with intellectual disabilities (SPECTROM) https://spectrom.wixsite.com/project (accessed on 12 September 2021) [19]. In this paper, we present the method used to develop CATS and its content, and compared its characteristics with other existing tools.

## 2. Materials and Methods

CATS was developed in two main stages. In stage 1, using a ‘bottom-up’ approach, one author (G.U.) interviewed 100 caregivers (both family and paid caregivers) of adults with ID who showed aggressive behaviour. During the interview, the caregivers were asked to describe what they thought were the triggers for aggressive behaviour in adults with ID they cared for. The interview scripts were analysed using a qualitative method, thematic analysis [35], which generated a list of themes for triggers. These themes were categorised under five headings. Several subthemes were created under each theme. In stage two, another author (B.L.) carried out a comprehensive literature review of publications on contextual trigger scales for challenging behaviour in ID and motivational analysis, which generated a pool of contextual trigger items. The themes from stage 1 and trigger items from stage 2 were combined and duplicates were removed. The final checklist of triggers was categorised using the same five themes that were used in stage 1. Both the themes in stage 1 and item checklists in stage 2 were ratified and confirmed by the third author (S.D.).

### 2.1. Stage 1

The work in stage 1 was done as part of a larger project called, ‘A Longitudinal Observational Study of Aggressive Behaviour in Adults with Intellectual Disabilities (ID)’. This project studied aggressive behaviour and its interventions prospectively during a period of 12 months among 100 adults with ID who were treated in psychiatric outpatient clinics in the West Midlands area of the UK [36]. The level of ID ranged from mild to profound, of which 23% participants had mild ID, 32% had moderate, 41% had severe, and 4% had profound ID; 41% of participants had a diagnosis of autism spectrum disorder and 4% was diagnosed with Asperger’s syndrome. Most participants had a comorbid medical/health condition and mental health diagnosis.

During the interview each caregiver (56% paid worker, 44% family carer) was asked ‘Do you know of any triggers for the aggression or what motivates the aggression?’ in the person that you care for. Caregivers’ responses were written down as close to verbatim as possible and to maintain anonymity, ‘P’ was used instead of names of individuals with ID (see Appendix B). The qualitative analysis of interview data looked for semantic patterns. Initial reading and re-reading of interview data helped to develop initial codes. Emergent themes were then analysed against the full data set to create thematic maps with candidate themes and subthemes. For inter-rater reliability, a trainee psychiatrist had independently analysed data and both S.T. and G.U. then discussed the final themes. In stage 2, these themes and subthemes were converted into a trigger items checklist.

### 2.2. Stage 2

In stage 2, a comprehensive literature review generated a pool of contextual trigger items that were then compared with the stage 1 themes/items and the duplicates were removed. Finally, the combined list of trigger items was categorised in contextual groups. A second author (S.D.) who is a senior professor of psychiatry in the field of ID ratified the final list for any duplication and missing items. Any disagreements on item categorisation were discussed and a final consensus was reached. The final draft of comprehensive checklist was then sent for feedback to 53 stakeholders involved in the SPECTROM project [19]. The stakeholders consisted of adults with ID and their families, researchers, direct support staff, and community learning disability team members including psychiatrists, speech and language therapists, a learning disability nurse, general practitioners and pharmacists, and representatives of service provider organisations.

The literature review was conducted using the following databases: OVID Journals (included Medline), PsycARTICLES, Embase, and PsycINFO. The search terms included: contextual scale OR rating scale OR functional assessment AND challenging behaviour OR problem behaviour AND intellectual disability.

The search terms were broad rather than specific to be inclusive and yield maximum results. Although we wanted to search for contextual triggers assessment scales, both contextual and functional assessment scales for challenging behaviour were included.

Articles published between 1985 and June 2019 in English language were searched. References cited in some relevant selected articles were screened to gather additional articles through cross-referencing. Articles relevant to the topic of interest were selected for the review, whereas articles that did not relate to the challenging behaviour scale were excluded. Conference abstracts, poster presentations, and scales unrelated to challenging behaviour were excluded. Scales related to quality of life, emotions, and mental health in people with ID were excluded.

Five trainees were interviewed to explore their experience of using CATS. Interviews were analysed using a thematic analysis method [35].

## 3. Results

**Figure 1:**
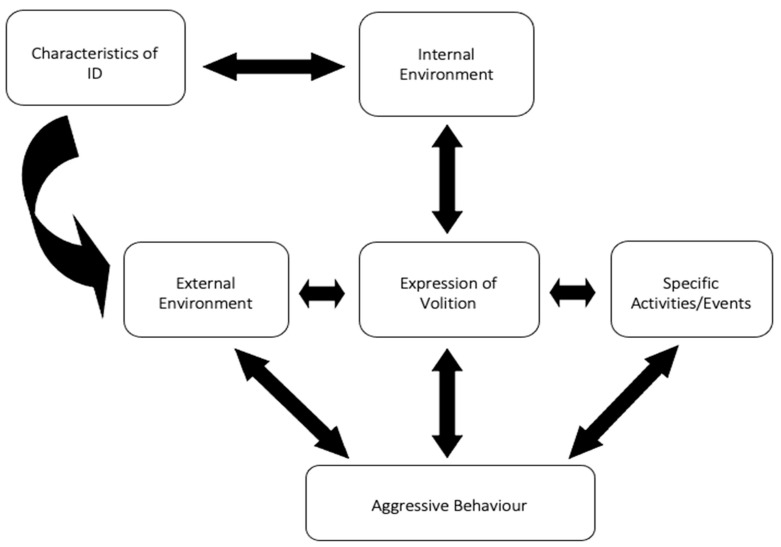
Schematic map of triggers and motivations for challenging behaviour identified by the caregivers [36].

In stage 1, 168 contextual variables were identified which were categorised under five main themes (internal environment (within the person), external environment (outside of the person), expression of volition, characteristics of ID, and specific activities/events) and 12 subthemes (Table 3). The highest number of items mentioned by the caregivers fell in the categories of external environment (*n* = 92), and internal environment (*n* = 76). Most commonly described subthemes by the caregivers were social environment, such as conflict with peers or witnessing conflicts and an issue around confrontation (*n* = 75), followed by limits to volition, such as not having demands met (*n* = 63); emotional state, such as too much excitement, stress, agitation, not feeling secured (*n* = 58); physical environment, such as noisy, busy, crowded environment overwhelming the person’s visual, auditory, and spatial stimuli, and making the atmosphere unpleasant or painful to bear (*n* = 54); and uncertainty (*n* = 54). There was overlap among the subthemes and contextual variables (Figure 1).

**Figure 2:**
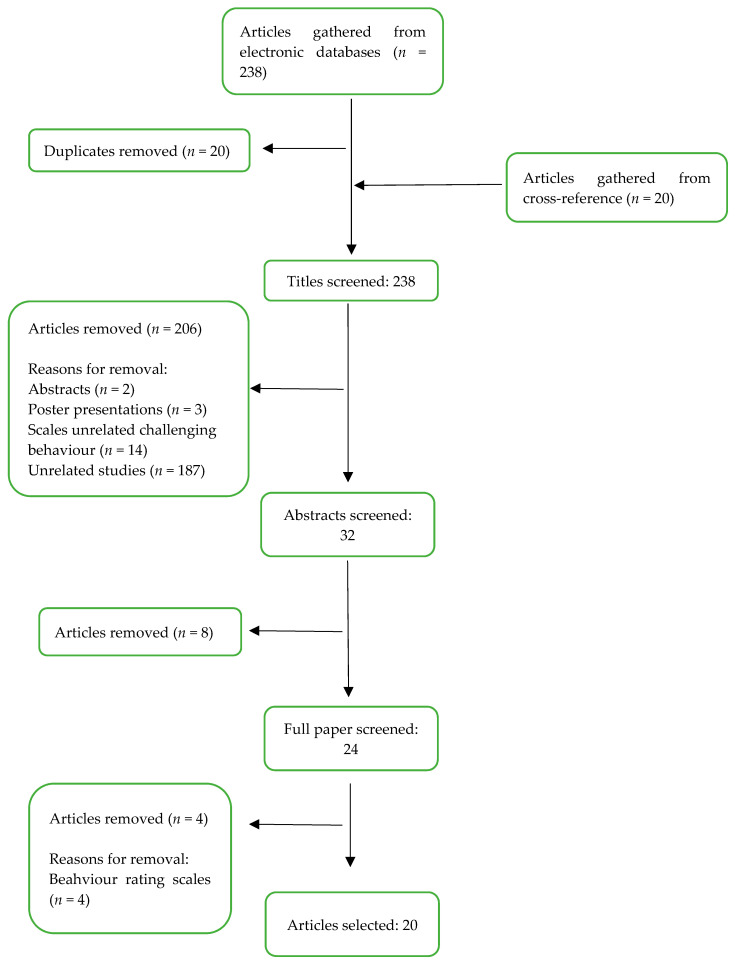
Preferred reporting items for systematic review and meta-analysis (PRISMA) flow chart of paper selection.

In stage 2, a total of 258 articles were gathered using the search, from which 20 articles were identified by cross-referencing and hand searching. Twenty articles were duplicates and removed. A total of 238 titles were screened from which 32 articles were selected for abstract screening. Reasons for exclusion can be found in Figure 2. Eight more articles were removed at the abstract screening stage as they did not contain information on scales/triggers of challenging behaviours. Twenty-four articles were selected for full text screening that focused on scales for challenging behaviours or provided information on scales. Four articles were further removed at full text screening as they were behaviour rating scales and not assessments for challenging behaviour. Finally, 20 articles were selected (see Figure 2) for inclusion in the review.

Scales related to functional analysis and assessment of contextual variables were given the same weight because useful triggers or motivations of challenging behaviours can be extracted from the functional analysis scales. For example, from the functional analysis checklist, for the question ‘Does the behaviour occur more in a crowded room?’, the context at which challenging behaviour will occur was extracted. In this case, the context ‘crowded room’ was extracted and placed in the item pool. Similarly, the context ‘making requests’ was extracted from the question ‘Does the behaviour occur when any request is made of this person?’ in the motivation assessment scale (MAS).

The SEC [16] items are not categorised, and the SEL [17], CHABA [18], and SEI [15] do not have subcategories. The lowest number of contextual variables (*n* = 17) was in the SEC (see Table 2). There are no scoring methods applied for these scales as they are checklists used to gauge information on contextual variables that act as triggers for challenging behaviour. Only CAIPB [13] has been tested for convergent and predictive validity. The effect size, using Cohen’s d, was 0.76 for log entries (convergent) and 0.85 for direct observation (predictive) [20]. CAIPB and CHABA have been tested for reliability. The Cronbach’s alpha for the total CAIPB scale was 0.95, and coefficients for the subscales ranged from 0.75 to 0.93. On the other hand, the coefficients for CHABA’s subscales varied between 0.65 to 0.87 [18]. SEI and CAIPB have been tested for inter-rater reliability. Inter-rater reliability of CAIPB can be considered good, with a mean intraclass correlation coefficient of 0.63 [21], while SEI can be considered excellent, with a median of 0.86 [15].

### 3.1. Generating Contextual Triggers Item Pool and Categorising Items

In this phase, items gathered from the literature review and from existing checklists were reviewed and included in the item pool. Over and above the contextual triggers identified at stage 1, further 183 items were added at stage 2 from the literature review data. Common contextual triggers within the item pool were then identified and a decision was made to remove, rephrase, or combine common items. The final number of trigger items included in CATS is 333 (see Appendix C). The items are rated as either present or absent.

### 3.2. Stakeholder Feedback on CATS

No major changes were made at this stage. CATS was found to be a comprehensive and helpful prompt for support staff to complete antecedent–behaviour–consequence (ABC) charts needed for functional assessment [10], as support staff often struggle to perceive the triggers for challenging behaviour.

All participants involved in the focus group interviews (*n* = 5) to evaluate SPECTROM [19] training found CATS, as a resource, useful and relevant to the trainees. One participant found it will be useful for agency workers who might not know the person with ID well enough to identify causes of challenging behaviour. Another mentioned that it is difficult to identify triggers of challenging behaviour and seeing trigger examples on paper can help caregivers understand what factors can trigger challenging behaviour in an adult with ID. One participant found CATS useful but stated that in his service only psychologists are allowed to identify triggers and assess challenging behaviour. Finally, the remaining two participants thought it was relevant to their work as they need to complete behaviour reports during an incident and record triggers for challenging behaviour.

## 4. Discussion

We developed a checklist called CATS for caregivers to identify triggers for challenging behaviour in adults with ID. CATS has 333 items categorised under five contextual categories; (a) external environment, (b) internal environment, (c) expression of volition, (d) characteristics associated with ID or autism, and (e) specific activities/events. Each of the main categories is further subdivided in many subcategories (Appendix C). Challenging behaviour is a means of communication and has a function [1]. Identifying the triggers will help to understand the issue that the person wants to communicate through her or his challenging behaviour. Inability to recognise triggers or antecedents can act as a barrier to functional assessment and development of intervention plans based on functional assessment and analysis. Therefore, CATS can be used to identify triggers/antecedents and aid understanding of function of behaviour and functional analysis. This would help to develop an intervention leading to a reduction in challenging behaviour and improvement in the person’s quality of life. This should subsequently help to reduce the staff’s own stress and burnout and improve their quality of life, thus setting a positive cycle.

### 4.1. CATS and Other Contextual Assessments

CATS, similar to other identified assessment of contextual triggers [13,14,15,16,17,18], considers items related to biological, physical, social/cultural, and instructional variables related to an activity or a task. However, a key difference is that for CATS we have chosen not to categorise items under these identified categories. Instead, the items related to biological variables have been categorised under medical conditions found under internal environment, while items related to social and physical variables have been categorised under external environment in CATS.

Triggers such as specific activities and events are missing in both CAIPB, and SEC. Specific activities especially relating to personal care may act as contextual triggers to challenging behaviour. Therefore, CATS includes a range of identified activities/events that may trigger challenging behaviour. However, it is important to note that many contextual variables/triggers are linked with each other, hence the categories and subcategories may overlap. For instance, personal care, which is a specific activity, may trigger aggression due to the nature of the task, which requires physical contact, which is an external, spatial trigger.

CATS provides information on the triggers for challenging behaviour that occurs at the present moment. CAIPB, in contrast, determines how likely it is that a person will display challenging behaviour based on the items scored using a 5-point scale from never to always. This means it is not specific to the challenging behaviour displayed at present time but rather identifies generic classes of contextual variables commonly associated with different challenging behaviours that an individual already presents. However, it may be difficult to remember triggers from the past, so some may be missed. SEC is completed prior to the manifestation of challenging behaviour, which may not happen on the same day the checklist is completed and thus, may be futile. Furthermore, SEC has only 17 items compared with 333 items in CATS. The SEL and SEI are similar to CAIPB, which does not provide information on triggers at the present moment.

### 4.2. Associations of Item to Challenging Behaviour

Medical and mental health conditions are known to affect challenging behaviour [37,38,39,40,41]. Of the 17 items in the SEC, only two cover medical conditions (menstrual period and appeared or complained of being ill) and no item on mental health. Of the 76 items in the SEL, two include mental health conditions (when depressed or sad and when tense or anxious), and three include medical conditions (around menstrual period, around seizures, and when ill). Of the 93 items in the CAIPB, two are on mental health (related to bipolar disorder and hallucinations) and three are on medical conditions (to specify acute and chronic illnesses, and menstrual discomfort). Of the 155 items in the SEI, only one item is related to medical conditions categorised under refusal category (client has complained of feeling unwell). CHABA has six medical items under the subheading ‘biomedical’ and seven psychological state items such as anger, unhappy, etc. under the subheading ‘emotional’, but no specific medical or psychiatric disorders. CATS has 54 trigger items on medical conditions and 10 on mental health issues, thus providing a much wider coverage of these issues compared with the existing scales.

Bowring and colleagues (2017) [42] found that being nonverbal or having limited understanding of communication were significantly associated with self-injurious behaviour, overall rate of challenging behaviour, aggressive–destructive behaviour, and stereotyped behaviour. Caregivers are not always aware of the better expressive (saying things) than comprehensive (understanding what has been said) skills that some people with ID may have. As a result, they may put inappropriate demands on the person that lead to challenging behaviour. In the SEI [15], SEL [17], and CAIPB [13], there are very few items (1–2 items) related to communication difficulties. CHABA [18] and SEC [16], in contrast, have no specific item relating to communication. CATS has 12 items on communication, including difficulties communicating and understanding speech.

Psychological traumas such as abuse may lead to challenging behaviour [1]. Therefore, this issue has to be identified and addressed to help reduce challenging behaviour. CATS includes triggers that occur in the present moment such as going to the dentist or feeling frightened, etc., along with triggers from the past, such as abuse a person may have experienced in the past. However, the existing scales do not include any items on abuse, thus neglecting the effects of psychological trauma on people with ID.

### 4.3. CATS and the Environment

CATS allows caregivers to carry out a broad assessment of a person’s social and physical context, and also consider medical and mental health needs when developing a person-centred positive behaviour support plan. Identification of triggers will help caregivers to understand the causes of challenging behaviour and adjust their support accordingly. This will subsequently reduce their stress from having to address challenging behaviour and will lead to a nonconfrontational approach. This itself will help to reduce the frequency and the severity of challenging behaviour.

McGill and colleagues [43] suggested 12 environments that should be capable to reduce challenging behaviour. For example, consistency in care and predictable routine are necessary to prevent challenging behaviour. These issues are covered in CATS under triggers associated with ID or autism, particularly problems with adaptability/uncertainty that might be causing challenging behaviour. CATS will help caregivers identify areas in the person’s environment that require improvement and build a capable environment for people with ID. This will help reduce challenging behaviour and improve the quality of life for a person with ID.

### 4.4. Strengths

CATS is much more comprehensive than the existing contextual assessment scales and caters for a range of contextual triggers that can be found in everyday life. CATS will act as an aide memoire and will facilitate the identification of triggers that caregivers may not have previously considered. This will help caregivers understand the reasons for challenging behaviour, help with the functional analysis, and aid in developing and implementing a person-centred intervention, such as a positive behaviour support plan. Functional assessment and analysis require training and are carried out by experts. Therefore, it is not available to caregivers and not always easy to implement by caregivers. Whereas CATS can easily be used personally by caregivers without any training, giving them a feeling of ownership of intervention programmes that might be developed using triggers identified by CATS assessment. This will also help with the implementation of the intervention. It is important to clarify that CATS will not replace functional analysis but will help with that process by identifying the antecedents of challenging behaviours. It can serve as an initial step towards a functional assessment of behaviour.

All participants involved in the focus group (*n* = 5) to evaluate SPECTROM [19] training found CATS, as a resource, useful and relevant to the trainees.

Existing scales were developed based on participants in residential settings and often data from children. The development of CATS involved adults with ID who lived in all types of community settings. The existing scales were developed by experts, whereas CATS was developed using a ‘bottom-up’ approach by interviewing caregivers and analysing interview data using a thematic analysis. This helps to provide a better face validity. The ‘bottom-up’ approach was complemented by a comprehensive literature review to add any missing items from the interview data.

### 4.5. Weakness

CATS has not been investigated for its applicability, acceptability, relevance, practicality, and psychometric properties such as reliability and validity. Although very comprehensive, CATS can still miss triggers that are not in the list. Given the comprehensive nature of the scale, caregivers may find the number of items to consider is overwhelming and time-consuming to complete, which may put them off from using the scale. However, we envisage that once the caregivers familiarise themselves with the scale, it will become easy for them to use CATS on more than one person and on a regular basis. Additionally, the layout of CATS with examples under each subcategory and coloured background should help with scoring. CATS can identify more than one trigger, thus not confirming the causality of the triggers for the challenging behaviour. To find a causal relationship, further functional analysis may be needed for hypothesis testing. In that way, CATS should be used to support functional behaviour analysis and not replace it. Similarly, the same or similar triggers may lead to more than one type of challenging behaviour or challenging behaviours in different settings and different time of the day/week. This may be confusing. Further research is needed to establish CATS’ psychometric properties and applicability, practicality, and relevance in day-to-day practice.

## 5. Conclusions

CATS is a comprehensive trigger checklist and can be used to assess challenging behaviour to identify triggers—contextual or antecedent variables that maintain challenging behaviours. The identification of antecedent variables can be used to assist functional assessment and develop an appropriate intervention. Any caregiver, either family members or paid support staff or nurses in hospitals, can use CATS without any prior training. CATS was developed using a stringent methodology that provides good face validity. However, its psychometric properties and validity need to be tested in a future study. In a recent field trial, CATS was found to be useful by a small number of staff who support adults with ID in community settings.

## Figures and Tables

**Table 1:** Scales and studies identified in the literature search.

	Scale Name	Studies Identified by Literature Search in Relation to Scale
**Scales related to contextual variables**	**Contextual Assessment Inventory for Problem Behaviour (CAIPB)**	McAtee (2002) [14] McAtee et al. (2004) [13] Carr et al. (2008) [20] Embregts et al. (2009) [21]
	**Setting Events List (SEL)**	McGill et al. (2005) [17]
	**Setting Events Checklist (SEC)**	Gardner et al. (1986) [16]
	**Setting Events Inventory (SEI)**	Tustin et al. (1997) [15]
	**Challenging Behaviour Attributions Scale (CHABA)**	Hastings (1997) [18]
**Scales related to functional assessment**	**Questions about Behavioural Function (QABF)**	Medeiros et al. (2013) [22] Watkins and Rapp (2013) [23] Paclawskyj et al. (2000) [24] Matson et al. (2012) [25] Singh et al. (2009) [26]
	**The Functional Analysis Checklist**	Sturmey (2001) [27]
	**Functional Analysis Screening tool (FAS)**	Iwata et al. (2013) [28]
	**Functional Assessment for Multiple Causality (FACT)**	Matson et al. (2003) [29]
	**Functional analysis**	Delgado-Casas et al. (2014) [30]
	**Motivation Assessment Scale (MAS)**	Durand and Crimmins (1988) [31] Singh et al. (1993) [32] Bihm et al. (1991) [33]

**Table 2:** Information on contextual/trigger scales.

Name of Scale	Items	Categories	Subcategories	Items per Sub/Category	Completion Method	Scale Development
Contextual Assessment Inventory of Problem Behaviour (CAIPB) [13]	80 items with 13 open ended questions	(a) Social/cultural, (b) Task/activity, (c) Physical and biological	Social/cultural category items were grouped into two subcategories: negative interactions and disappointment. Tasks/activities category items were grouped into two subcategories: factors related to tasks and factors related to daily routines. Physical category items were grouped into two subcategories: uncomfortable environment and changes in environment. Biological category items were grouped into three subcategories: medication, illness, and physiological states.	**Social/Cultural**— Negative interactions: 22 items and 1 open-ended question; Disappointments: 10 items and 2 open-ended questions. **Tasks/Activities**— Factors related to tasks: 13 items and 1 open-ended question; Factors related to daily routines: 8 items and 2 open-ended questions. **Physical**— Uncomfortable environment: 6 items and 1 open-ended question; Changes in environment: 5 items and 2 open-ended questions. **Biological Category** Medication: 2 items and 1 open-ended question; Illness: 4 items and 1 open-ended question. Physiological states: 10 and 2 open-ended questions.	Likelihood of association between challenging behaviour (CB) and items are rated on a 5-point scale, from 1 (never) to 5 (always).	Scale development included 6 steps: review of the existing literature, generation of item pool, group items into the four generic categories, identify subcategories, determine format for measurement, administer inventory to staff, and evaluating the inventory.
Setting Event Checklist (SEC) [16]	17 items	No categories	No subcategories	Not applicable	Occurrence/nonoccurrence of each item in the morning (a.m.) or evening (p.m.) recorded prior to attending programmes.	Developed by discussing with residential staff regarding situations or events that would provoke CB for a group of individuals with moderate and severe intellectual disabilities who lived in the residential facility and presented chronic CB while attending a community work training program.
Setting Event List (SEL) [17]	76 items	(a) Physical setting, (b) Time of day, (c) Day of week, (d) Time of year, (e) Weather conditions, (f) Activities, (g) Social contexts, (h) Personal contexts, (i) Presence of particular clients, (j) Presence of particular staff members	No subcategories	(a) Physical setting: 12 (b) Time of day: 9 (c) Day of week: 7 (d) Time of year: 6 (e) Weather conditions: 6 (f) Activities: 13 (g) Social context: 9 (h) Personal context: 14 (i) Presence of particular clients and presence of particular staff members; Number of items equivalent to other clients and staff.	Report whether target CB was more or less likely (or ’makes no difference’ or ’not applicable‘) across the items.	Developed in reference to 22 individuals with intellectual disabilities in 18 different residential service settings.
Setting Events Inventory (SEI) [15]	155 items	(a) Carer Attention, (b) Carer Instruction, (c) Carer Touch, (d) Criticism, (e) Correcting Consequence, (f) Peer Agitation, (g) Peer Encroachment, (h) Possessions, (i) Task Difficulty, (j) Environmental Noise, (k) Organised Activity, (l) Changes, (m) Disappointment, (n) Fine Motor Activity, (o) Physical Activity, (p) Low Participation, (q) Refusal, (r) Seeking Support and (s) Disruptive Communication	No subcategories	(a) Carer attention: 12 (b) Carer instruction: 11 (c) Carer touch: 8 (d) Criticism: 10 (e) Correcting consequence: 9 (f) Peer agitation: 13 (g) Peer encroachment: 11 (h) Possessions: 5 (i) Task difficulty: 4 (j) Environmental noise: 3 (k) Organised activity: 15 (l) Changes: 15 (m) Disappointment: 7 (n) Fine motor activity: 4 (o) Physical activity: 5 (p) Low participation: 6 (q) Refusal: 8 (r) Seeking support: 6 (s) Disruptive communication: 3	Rate the likelihood of challenging behaviour occurring within 5 min of the client encountering each situation using a four-point scale: 0 (unlikely or occurs on less than 10% of occasions), 1 (sometimes or between 11% and 50% of occasions), 2 (often or from 51% to 80% of occasions), and 3 (almost always or over 81% of occasions).	Developed based on available literature and on information about individual clients that had been gathered using antecedent–behaviour–consequence (ABC) charts by the researcher.
Challenging Behaviour Attributions Scale (CHABA) [18]	33 items	(a) Learned positive behaviour (LP), (b) Learned negative behaviour (LN), (c) Biomedical (BM), (d) Emotional (EM), (e) Physical environment (PE), (f) Stimulation (ST)	No subcategories	(a) LP: 3 (b) LN: 3 (c) BM: 6 (d) EM: 7 (e) PE: 8 (f) ST: 6	Five-point scale ranging from ‘very unlikely’ to ‘very likely.’	Further developed from a 25-item scale previously developed by Hastings et al. (1997) [34] in which the items were collated from literature review and followed by ratings on the items by support staff based on case vignettes.

**Table 3:** Prevalence of themes and subthemes detected at stage 1 [36].

Theme/ Subtheme	Number of Carers Mentioning Theme (*n* = 100)
External Environment	**92**
Physical Environment	54
Social Environment	75
Internal Environment	**76**
Aversive Physical States	22
Medical Conditions	28
Mental Health Problems	15
Emotional States	58
Expression of Volition	**65**
Goal-Directed Behaviour	8
Limits To Volition	63
Characteristics of ID	**57**
Problems With Adaptability/Uncertainly	54
Communication Difficulties	16
Specific Activities/Events	**26**
Specific Activities	16
Specific Events	13

**Figure A1:**
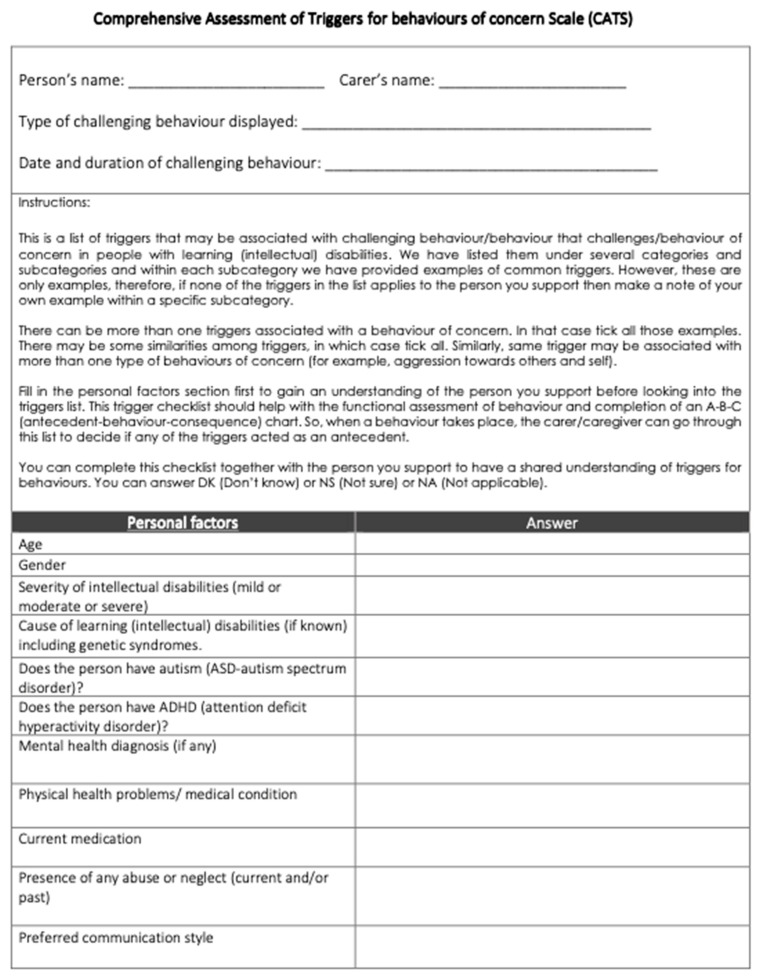

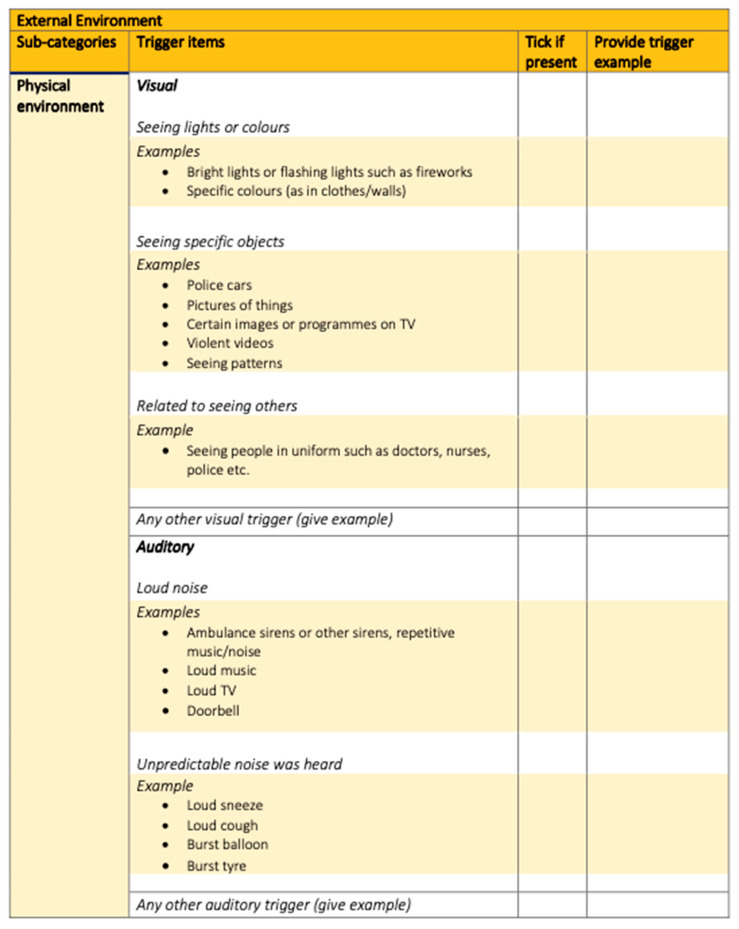

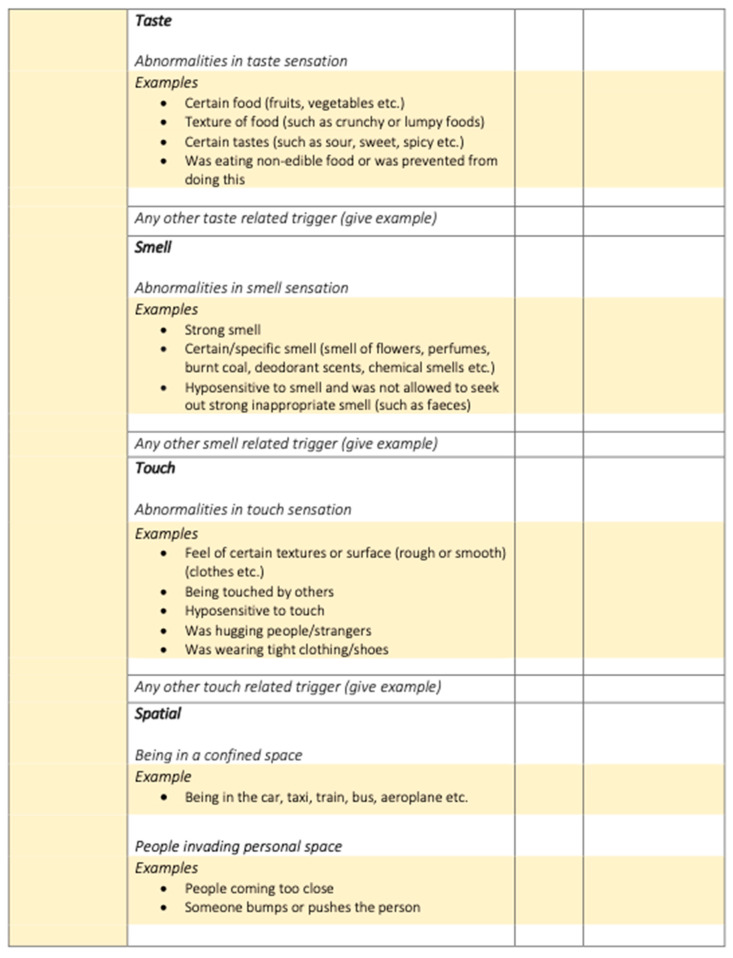

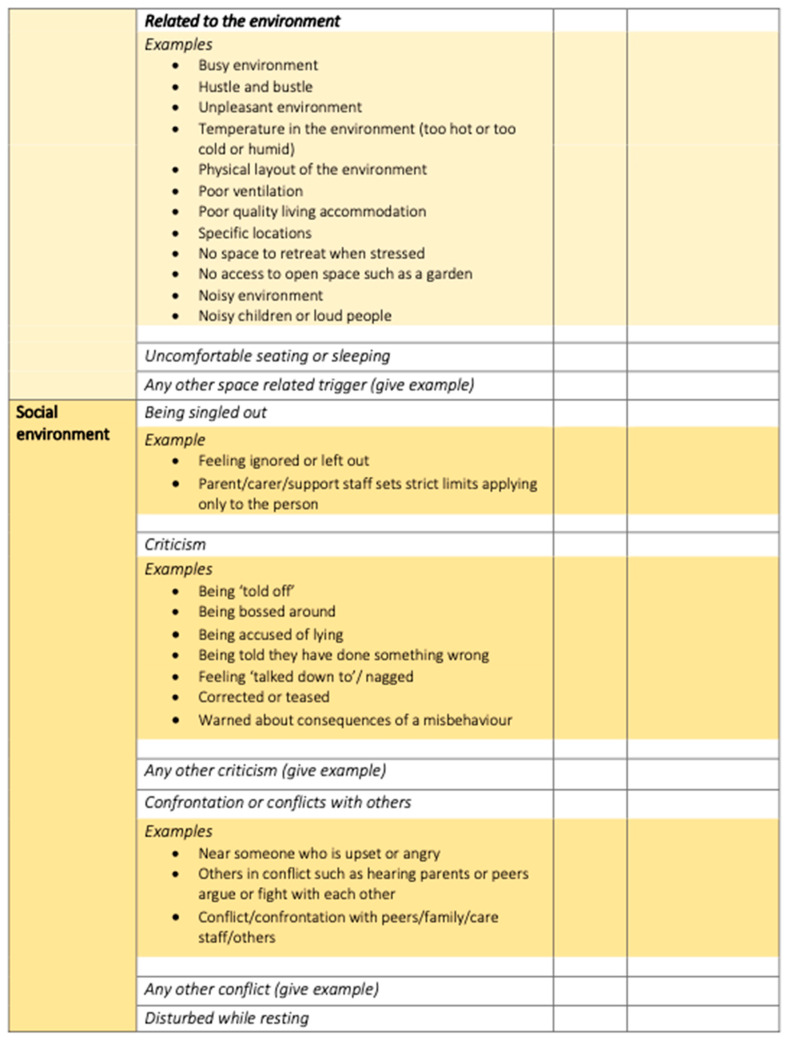

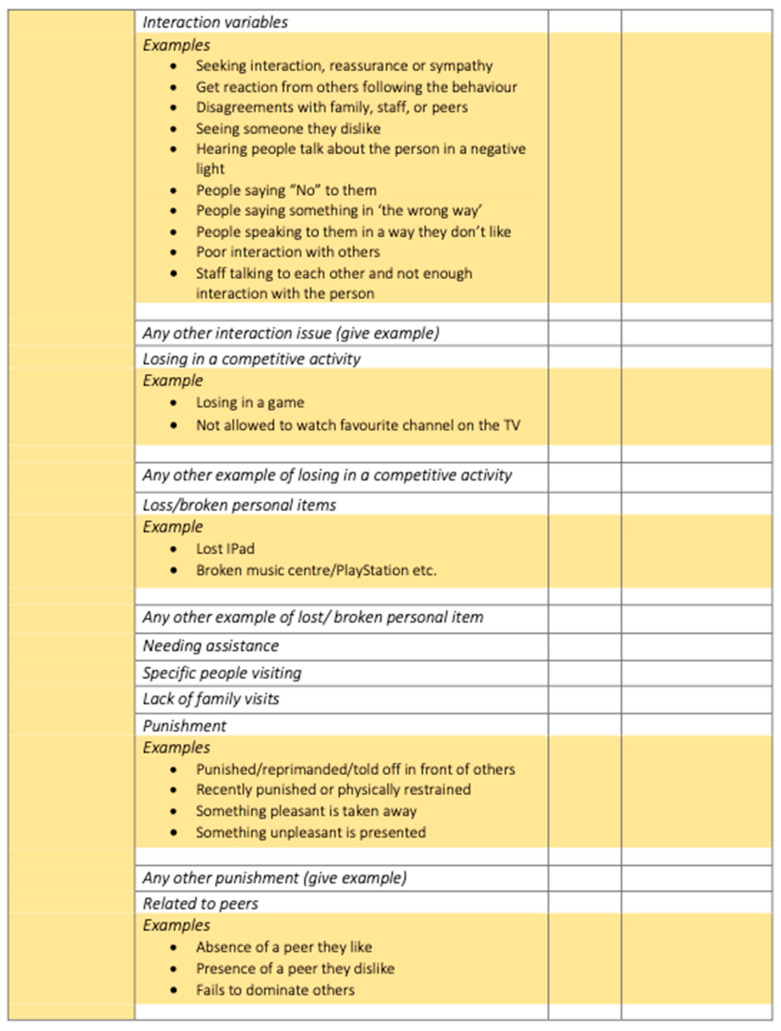

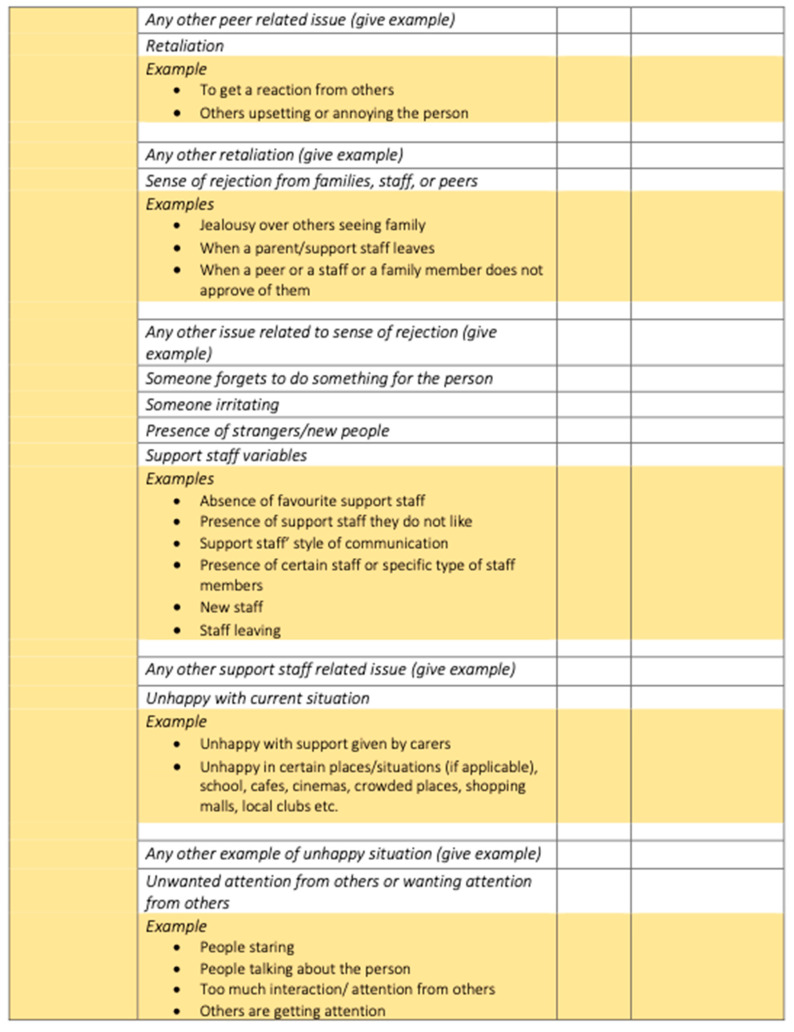

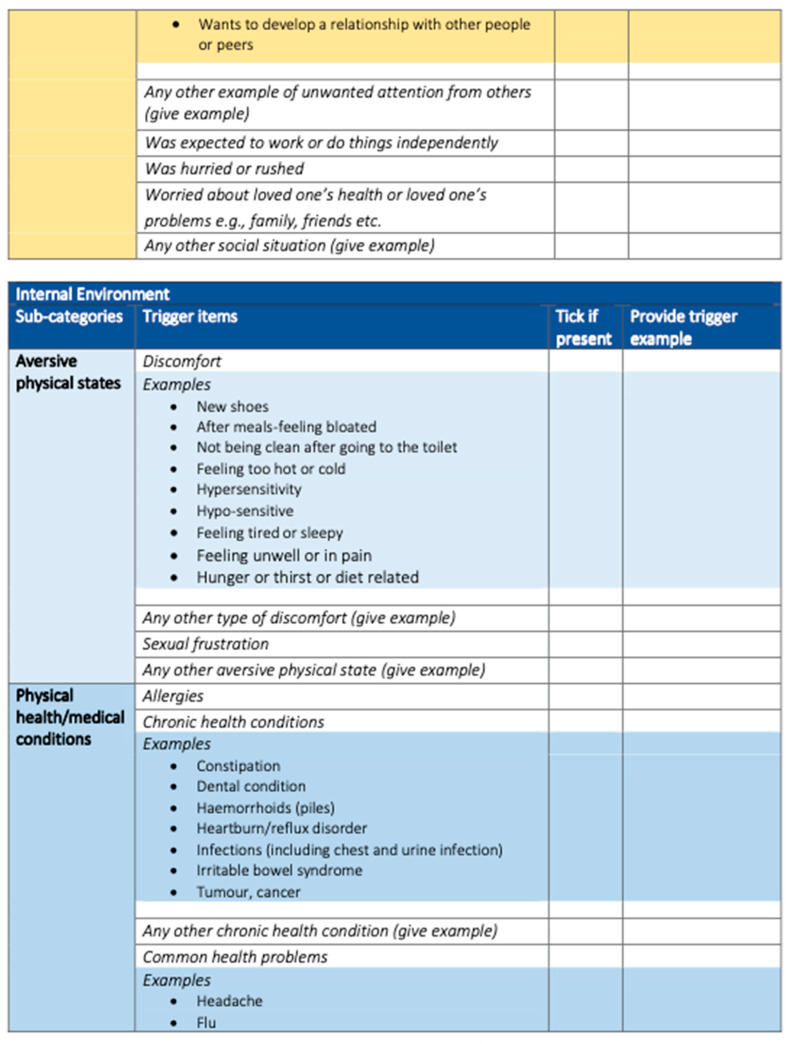

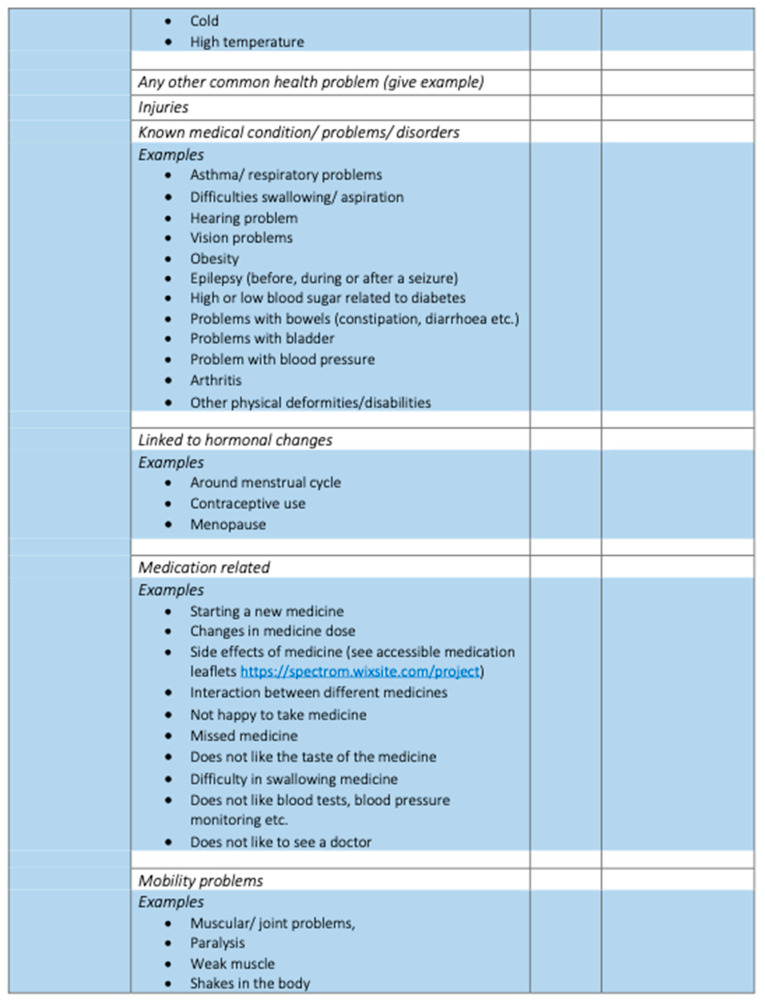

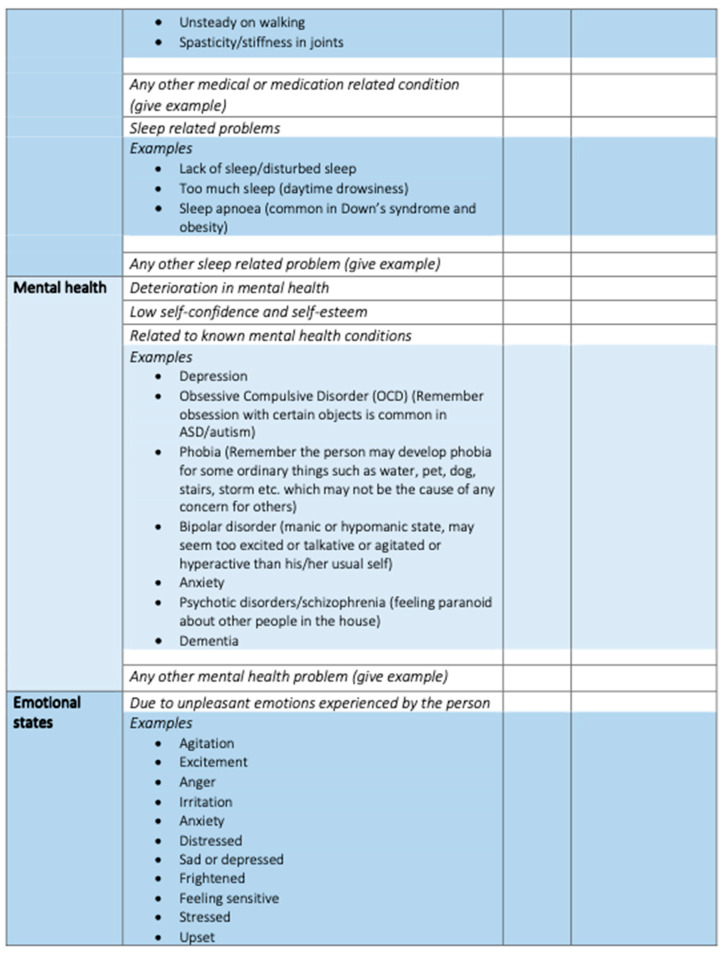

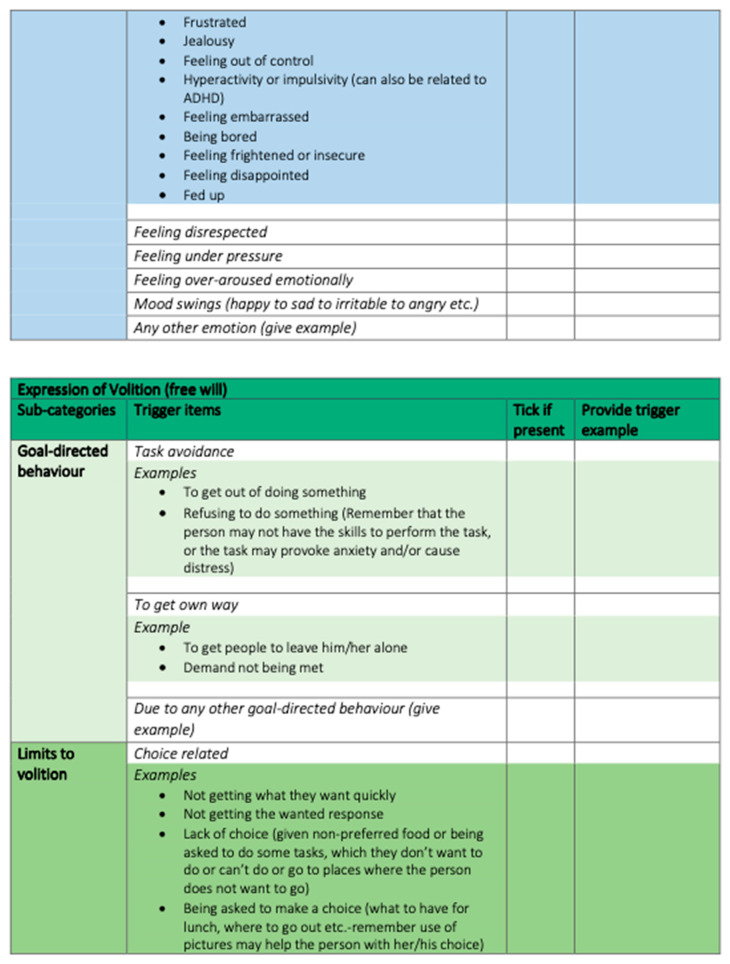

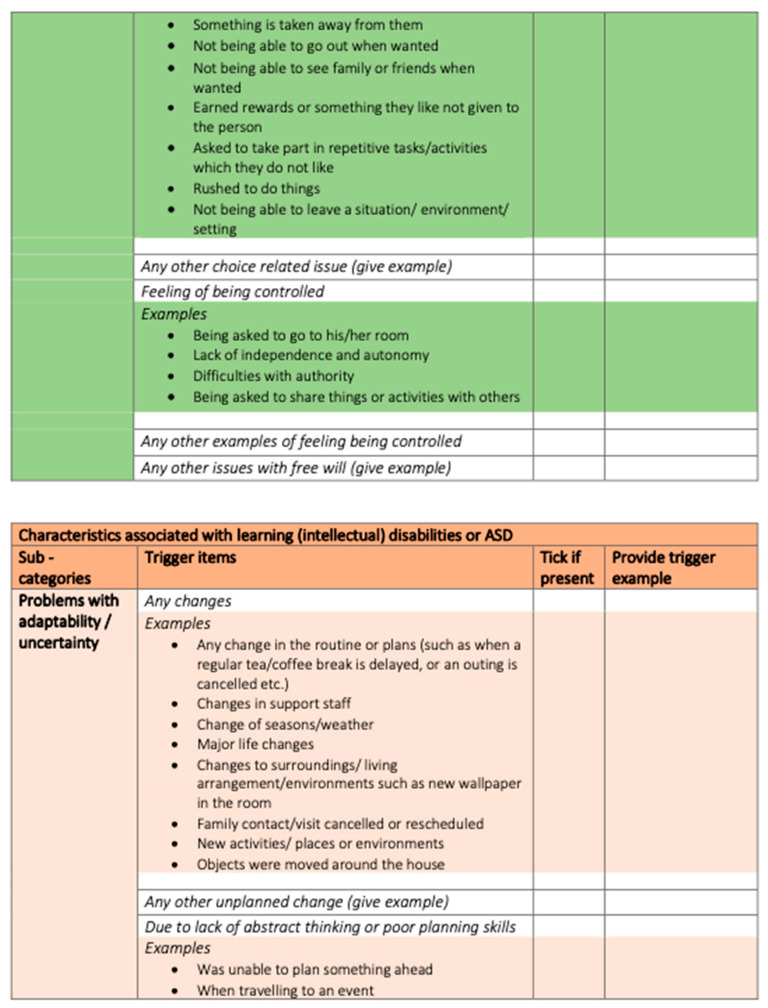

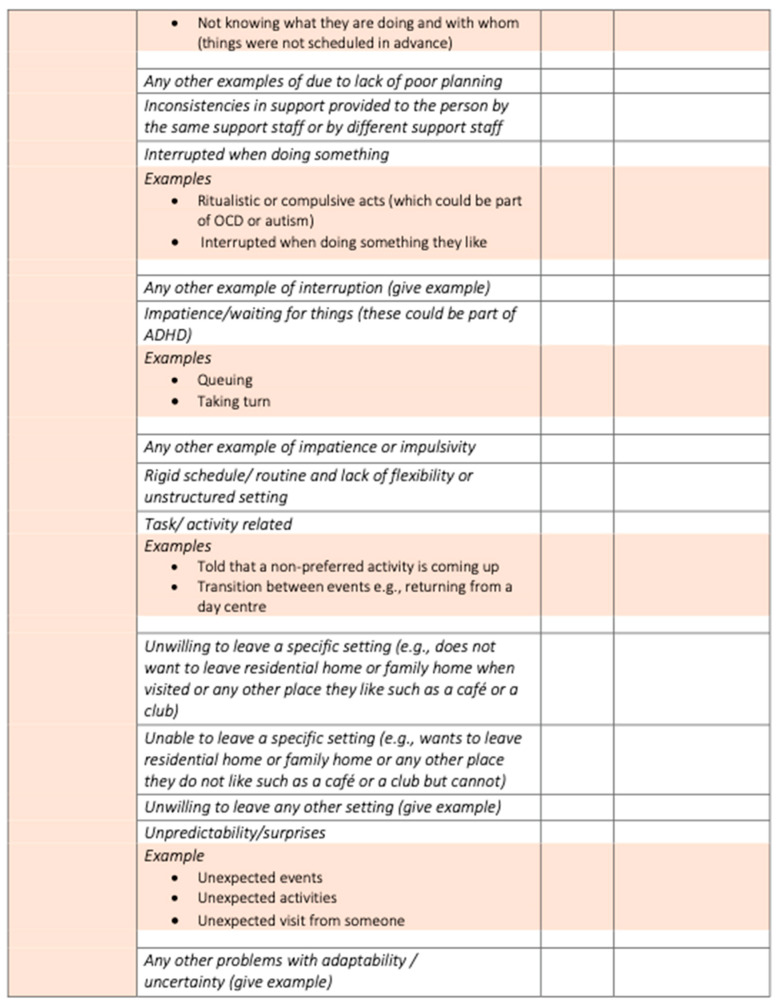

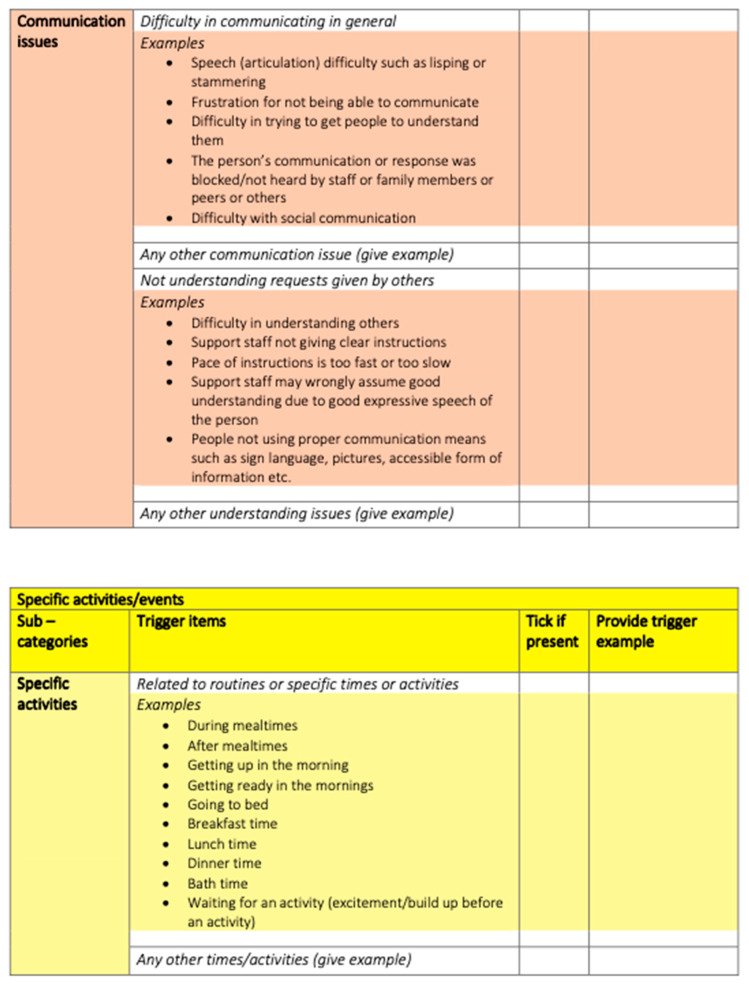

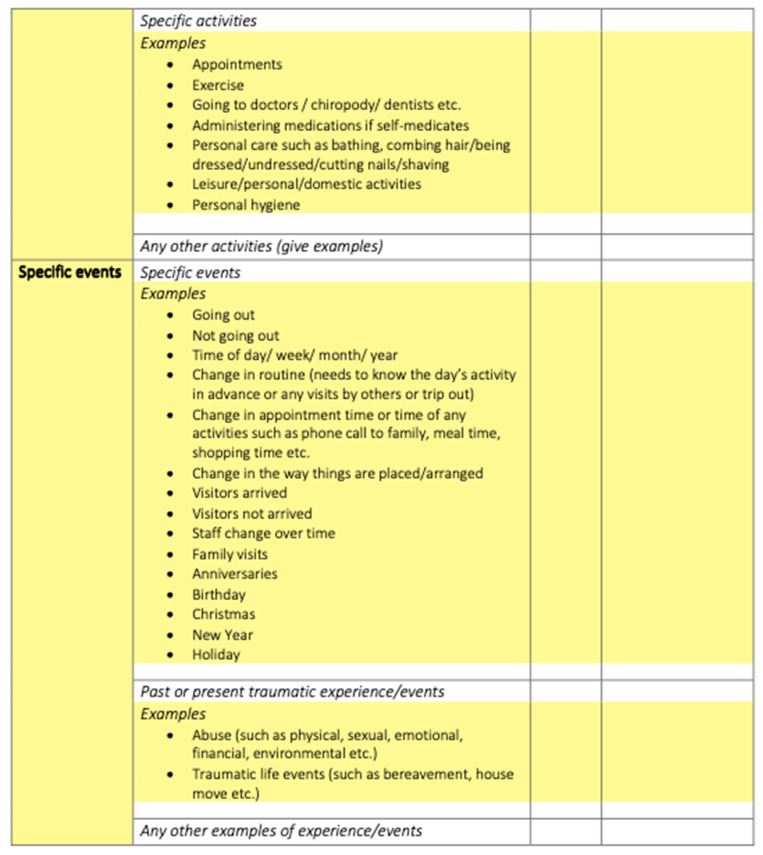
Comprehensive Assessment of Triggers for behaviours of concern Scale (CATS).

## Data Availability

Data supporting reported results from stage 1 can be found at https://etheses.bham.ac.uk/id/eprint/4735/1/Unwin14PhD.pdf (accessed on 12 September 2021).

## Appendix A

**Table A1:** BMPPS model of challenging behaviour.

Behaviour (B)	A list and clear description of the target behaviour(s) to be addressed.
	The type and the nature of the behaviour(s).
	Past history of similar behaviour.
	Baseline behaviour prior to the onset of current problem behaviour.
	The onset of the behaviour(s) to describe whether they appeared gradually over time or relatively abruptly perhaps precipitated by an acute event.
	The frequency, severity, and duration of the behaviour(s).
	Reactions to the behaviour by the person/ others/ services.
	Associated behaviours (other relevant behaviour than the target behaviours).
	The impact of the behaviour(s) on the person’s life, other’s life, and the environment.
	Consequences of problem behaviour e.g., reduced quality of life for the individual and her/his caregivers; reduced access to services including education, day service, and employment opportunity and may lead to a threatened or actual loss of placement in a residential setting or day placement; reduced social activities including leisure activities, access to friends, etc.; physically restraint; medicated; in severe cases, hospitalised or prosecuted.
	Assessment of risks of behaviour e.g., risk to others; risk to the individual; risk to the environment and other risks.
	Previous risk assessment.
	Review of previous and current measures taken to reduce risks to assess their effectiveness.
	The function of the behaviour (what does the behaviour want to achieve?).
Medical and Organic Factors (M)	Physical symptoms (toothache, tummy ache, heartburn, headache etc.).
	Acute or chronic physical /medical conditions (cardiovascular, respiratory, endocrine, gastrointestinal, musculoskeletal, dental, skin and genito-urinary).
	Physical disabilities.
	Problem with sleep, appetite, weight, bowel, bladder.
	Epilepsy, and other neurological conditions (spasticity, movement disorders, multiple sclerosis, brain tumour etc.).
	Genetic conditions (Lesch-Nyhan syndrome, Prader-Willi syndrome, Fragile X syndrome, Smith-Magenis syndrome etc.).
	Sensory impairment.
	Communication/speech problems.
	Drug and alcohol related factors.
	Current medication, previous medication, polypharmacy and high dose medication use, adverse effects including anticholinergic burden.
Relevant histories (Person)	Family, occupational, relationship.
	Current accommodation, daytime occupation, leisure activities, family circumstances.
	Patient’s interests, strengths-abilities, likes, dislikes and preferences and how they express these opportunities, impact of disabilities, needs (including mental and physical health), and service and resource gaps.
	Their history-social, developmental, psychological and history of use of services.
	Difficulties in developing fulfilling relationships.
	Daily/ weekly diary.
Psychological/ Psychiatric Factors (P)	Psychiatric Disorders: Psychoses, Bipolar disorders, dementia, Depressive disorders, and anxiety related disorders etc.
	Psychological/ emotional issues (such as bereavement, recurrent stress and relationship difficulties leading to loss of self-esteem and isolation, abuse, etc.). New/ ongoing/ recurrent stress. Difficulty in developing fulfilling relationships. Developmental disorders, like Autism Spectrum Disorder (ASD) and Attention Deficit Hyperactivity Disorder (ADHD), including impulsivity, Neuropsychological factors.
	Relevant history of psychological development.
	Psychological symptoms: depression, anxiety etc.
	Personality traits.
	Dysregulated arousal and affect.
Social/environmental Factors (S)	Crowded/noisy/uncomfortable environment.
	Demanding activities, lack of interesting activities, too many changes in the activities etc.
	Personalities of other people/ staff and interactions with other people.
	Change in the environment, activities of daily living at home (e.g., washing, cleaning), activities of daily living outside home (e.g., shopping), relationships, influence of life events, occupation and activities including leisure activities and financial situation, therapeutic interventions.
	Under- or over-stimulating environment.
	Issues relating to integration within the wider society, stigmatisation and discrimination.
	Carer issues, including levels of stress and lack of support for carers.
	Changes required in the level of supervision and support, major life events including abuse.
	Adequate support for patients and also their caregivers (both family caregivers and paid care staff).

## Appendix B

**Table A2:** Quotes to illustrate categories of triggers/motivations.

Theme/ Subtheme	Illustrative Quotes
**External Environment**	
Physical environment	P does not like loud people, the noise will agitate him. People being noisy. P does not like noisy environments. Excessive noise. Loud, unexpected noises, sneezing, coughing. Loud noises will make P jump. P gets verbally aggressive and cross when there are loud, unexpected noises [for example], fireworks. Things on TV, favourite shows like Casualty and Holby City: gruesome things and seeing blood on TV. Violent videos, P would watch them over and over again and get wound up and aggressive himself. P would be aggressive when asked to turn them off. Busyness around the home. P lives in a large group home, and it is generally quite busy and noisy; P does not tolerate this very well. Staff changeover: this is because it is a busy time in the house with lots of noise. P does not like crowds or being in groups. P does not like being in busy places, large shopping centres. He will start pacing and shouting as he becomes more distressed in his surroundings. People invading her personal space—P will tend to lash out. P does not like being touched. P does not like too much sensory input and can become overloaded which leads to anxiety and agitation.
Social environment	P will act out to become the centre of attention. P will physically attack other residents to become the centre of attention. P will have temper tantrums to seek company and attention. This is a learnt behaviour as P knows it will get her what she wants—attention from someone. Seems P is always looking for a reaction, for example, his banging will escalate more and more until he has been told to stop it. P likes to get a reaction. To provoke negative reactions in other people, especially other service users. This is part of P exerting his power and authority over people. Being ignored or observing others getting all the attention. Jealousy over other residents getting more attention from staff. Jealousy over other service users especially around staff attention, going out and family visits. P does not see her family very often and she can get jealous when other residents see their family and go to stay with them... this can lead to aggression. When relatives visit, approximately twice a year—exacerbates the problem as P gets excited. Family visits: P can become anxious when he knows his family are due to visit. Going to his parents; P’s last severe outburst was related to the build-up in agitation before he went home. Family contact—pre and post; P seems to get upset around family visits, could be because she feels rejected. Family rejection can be a key trigger; because of this, P can interpret a lot of things as rejection and this then triggers her aggressive behaviour. Conflicting with his father; they have physical fist fights and tend to wind each other up. Friction with another resident exacerbates the aggression. P is always verbally aggressive towards this other resident but will take out anger on the other residents as well. P clashes with another service user; the other person is very able and verbal as well. They fight over the ‘pecking order’ in the house but do sometimes get on. Two certain staff members—P will be very verbally aggressive when they are on shift. There have been frictions with certain members of staff and the way they supported P. People disagreeing with P—you are better off agreeing with some things. Being reminded he has done something wrong. Being caught out when he has done something wrong. When she is accused of lying. Criticism and being corrected. Other people’s behavioural problems [for example], screaming or anxiety-related behaviours. P does not like to sense other people’s anxiety as it upsets him. Verbal aggression—if other service users are shouting. P gets upset when he hears his parents arguing.
**Internal Environment**	
Aversive physical states	When P is hungry or thirsty. When poorly. When tired. When P gets too hot. Pain; most of P’s behaviour is related to pain. Pain, hence paracetamol prn [pro re nata/as needed] is often effective as a first line intervention.
Medical conditions	The menopause. PMT [pre-menstrual tension]. UTIs [urinary tract infections]. Hyper or hypo related to diabetes. Constipation and irritable bowel syndrome. Epilepsy. Leading up to a seizure.
Mental health problems	P’s mental health—P will be more irritable and more inclined to shout and slam doors when she is in a hypomanic phase. Paranoia is P’s key trigger; thinking others are talking/spying/staring or looking at him. P can be very violent when his paranoia is bad. Increase in schizophrenia symptoms leads to increase in aggression. When P is depressed, she will be more likely to self-injure. SIB related to depression.
Emotional states	Excitability can lead to an outburst as P gets mixed up with his emotions. P can get anxious when he gets really excited; the increasing emotion can get misinterpreted. The build up, anticipation and excitement of activities can lead to aggression. P will become anxious when waiting for an activity. Things that cause anxiety, then agitation, then they can cause aggression. Behaviour is anxiety related—all to do with this. Anxiety; all of P’s behaviours are anxiety related. This leads to frustration and then possibly aggression. P will act out when bored. P does not tolerate boredom—P can be aggressive when bored. Boredom—P needs to be kept busy and needs things to look forward to. Feeling he has made a fool of himself. P gets annoyed with himself if he thinks he has made a fool of himself, if someone bumps into him, he will say sorry and be cross with himself. P also gets really upset if he feels like he has caused a fuss. Some days P can be very sensitive. P gets wound up easily and worries over people talking about her.
**Expression of Volition**	
Goal-directed behaviour	A lot could be learned behaviour as it is very effective for P as he gets what he wants. Can be task avoidance—will shout to get out of doing something. To get his own way. To get out of doing things.
Limits to volition	Usually because P wants something and is unable to get it quickly enough or not at all—demands not being met. Demands must be met immediately, if P cannot find a video he wants, this can be a trigger. Requests being turned down, P not getting what he wants straight away and demands not being met. When P thinks he is going out or wants to go out and is told he is not going out; when other residents get to go out but he does not. P will go and put his coat on and say is he going out but will spit and shout and scream when told he is not. Not going out. A lot of P’s behaviours centre around going out—wanting to and not being able to. There are tensions in the evening with P wanting to go out. Aggression tends to occur when demands are not met—usually around drinks. Not getting chocolate when he wants it. Saying ‘no’ or P being stopped from doing what he wants to do. Encouraging P to do more, especially activities, when he does not want to. Being asked to do something he does not want to—feeling that he is being nagged.
**Specific Activities/Events**	
Specific activities	Personal care, especially combing P’s hair; six out of every seven mornings, P is difficult. P’s physical aggression is often around her personal care in the morning. If P feels he is being interfered with—issues around personal care, however, staff have a duty of care to P so this is unavoidable. Encouraging P to take his medications. P will get agitated and aggressive when going to the doctors or dentists. Chiropody visits. Build up to special occasions, visits, appointments, et cetera.
Specific events	Christmas can be very stressful for P; there is often a build up of angry and loud behaviour. P has expectations and sees others getting presents and gets jealous. Christmas is a difficult time for P; this can increase her aggressive behaviour. P will throw decorations off the tree et cetera—largely due to the excitement of it all, not intentionally aggressive. Christmas—Christmas holidays; P will get excited and then anxious. Other events have this effect as well, even day trips out. P will repetitively ask what day Christmas is and what is happening.
**Characteristics of ID**	
Problems with adaptability/uncertainly	Changes to routine, feeling things are out of his control. Changes to routine—P needs to be prepared, for example, if someone is calling at the house, otherwise P would start spitting at them. Unexpected changes in staff—when P not informed of these. When things are late or not going to plan—everything has to have a time, for example, when a phone call is late, P will chunter and bang things et cetera. People not keeping appointments or keeping P waiting; this used to be a problem in the past but is less so now. Uncertainly around change—P needs to know what she is doing every day. Anxiety around feeling like he does not know what will happen next. Changes to routine, this causes anxiety. This is P’s key trigger. Everything has to have its own place. Someone moving P’s belongings, especially in her room. P does not like change very much [for example], changes to furniture in home whilst she is away at college. Cancelled activities could be a big trigger for P. However, if P is provided with plenty of information, verbally and pictorially, and an alternative activity provided, then P can avoid getting anxious.
Problems with Communication	Feeling like he has not been understood or someone telling P they have not understood him. People not understanding P and therefore leading to frustration and aggression. When P does not understand what others are saying. P’s aggression is communicative. To indicate a need – due to communication difficulties; P cannot say what she needs or wants.
**Predictability of Behaviour**	
Behaviour is unpredictable	Currently, there are often not the obvious triggers that there used to be. The behaviour is becoming more unpredictable. P is having unpredictable mood swings—P can be very happy and content one minute and then he might lash out for example, he hit another service user three times across the face. P can be very unpredictable. Outbursts are often unprecedented. P has random outbursts. Often [you] cannot find a trigger. Really varied, hard to tell what the trigger is. Sometimes it is not always clear what sparks the behaviour.
Behaviour is predictable	P is not usually aggressive for no reason. When P has been unhappy and angry, there is always a reason.
